# Pharmacological manipulation of peroxisome proliferator-activated receptor γ (PPARγ) reveals a role for anti-oxidant protection in a model of Parkinson's disease

**DOI:** 10.1016/j.expneurol.2012.02.017

**Published:** 2012-06

**Authors:** Heather L. Martin, Ross B. Mounsey, Sarah Mustafa, Kinnari Sathe, Peter Teismann

**Affiliations:** School of Medical Sciences, University of Aberdeen, Institute of Medical Sciences, Foresterhill, Aberdeen, AB25 2ZD, UK

**Keywords:** BSO, buthionine-sulfoximine, DCF, 2′7′-dichlorofluorescein, DCF-DA, 2′7′-dichlorofluorescein diacetate, DOPAC, 3,4-dihydrophenylacetic acid, GFAP, glial fibrillary acid protein, GST, glutathione-S-transferase, Iba1, ionized calcium-binding adaptor molecule 1, LDH, lactate dehydrogenase, MPP^+^, 1-methyl-4-phenylpyridinium, MPTP, 1-methyl-4-phenyl-1,2,3,6-tetrahydropyridine, MTT, 3-(4,5-dimethylthizol-2-yl)-2,5-diphenyltetrazolium bromide, PD, Parkinson's disease, PPARγ, peroxisome proliferator-activated receptor γ, ROS, reactive oxygen species, SNpc, substantia nigra pars compacta, SOD, superoxide dismutase, TH, tyrosine hydroxylase, Parkinson's disease, Peroxisome proliferator-activated receptor γ, MPTP, MPP^+^, Neurodegeneration

## Abstract

Peroxisome proliferator-activated receptor γ (PPARγ) agonists have been shown to provide neuroprotection in a number of neurodegenerative diseases including Parkinson's disease and Alzheimer's disease. These protective effects are primarily considered to result from the anti-inflammatory actions of PPARγ, however, there is increasing evidence that anti-oxidant mechanisms may also contribute. This study explored the impact of the PPARγ agonist rosiglitazone and the PPARγ antagonist GW9662 in the MPP^+^/MPTP (1-methyl-4-phenylpyridinium/1-methyl-4-phenyl-1,2,3,6-tetrahydropyridine) model of Parkinson's disease, focussing on oxidative stress mechanisms. Rosiglitazone attenuated reactive oxygen species formation induced by MPP^+^ in SH-SY5Y cells concurrent with an upregulation of glutathione-S-transferase activity, but not superoxide dismutase activity. These responses were not attenuated by cotreatment with GW9662 suggesting that PPARγ activation is not required. The localisation of PPARγ *in vivo* to dopaminergic neurons of the substantia nigra pars compacta (SNpc) was established by immunohistochemistry and PPARγ levels were found to be upregulated 7 days after MPTP treatment. The importance of PPARγ in protecting against MPTP toxicity was confirmed by treating C57BL6 mice with GW9662. Treatment with GW9662 increased MPTP-induced neuronal loss in the SNpc whilst not affecting MPTP-induced reductions in striatal dopamine and 3,4-dihdroxyphenylacetic acid. GW9662 also caused neuronal loss in the SNpc of saline-treated mice. The evidence presented here supports the role of anti-oxidant mechanisms in the protective effects of PPARγ agonists in neurodegenerative diseases, but indicates that these effects may be independent of PPARγ activation. It also demonstrates the importance of PPARγ activity for neuronal survival within the SNpc.

## Highlights

► We investigate the role of PPARγ in a model of Parkinson's disease. ► Rosiglitazone has PPARγ independent anti-oxidant effects. ► Rosiglitazone involves upregulation of glutathione-S-transferase activity. ► PPARγ is upregulated after the neurotoxin MPTP. ► PPARγ antagonism is toxic to neurons in the SNpc.

## Introduction

Peroxisome proliferator-activated receptor γ (PPARγ) is a ligand activated transcription factor with a wide variety of biological roles including control of lipid metabolism and regulation of inflammation (Delerive et al., 2001; Desvergne and Wahli, 1999; Devchand et al., 1996). Inflammation is an important feature in the pathogenesis of a whole spectrum of neurodegenerative diseases (Glass et al., 2010), consequentially PPARγ agonists have the potential to provide neuroprotective effects. Protective effects have been seen with PPARγ agonists in murine models of Alzheimer's disease (Escribano et al., 2009), Parkinson's disease (PD) (Dehmer et al., 2004; Breidert et al., 2002; Schintu et al., 2009; Carta et al., 2011), amyotrophic lateral sclerosis (Kiaei et al., 2005), Huntington's disease (Quintanilla et al., 2008) and multiple sclerosis (Feinstein et al., 2002; Niino et al., 2001). These effects are primarily considered to be a result of the anti-inflammatory actions of PPARγ. However, increases in anti-oxidant gene expression (Nicolakakis et al., 2008) and modulation of cholesterol metabolism (Xiong et al., 2008) are also thought to contribute to the positive effects seen in Alzheimer's disease models. Indeed PPARγ has been shown to be important for the regulation of a number of anti-oxidant genes including glutathione-S-transferases (Park et al., 2004), Cu/Zn superoxide dismutase (SOD1) (Yoo et al., 1999) and catalase (Girnun et al., 2002).

Parkinson's disease is a common neurodegenerative disease that is characterised by loss of the dopaminergic neurons of the nigrostriatal pathway (Dauer and Przedborski, 2003). The pathogenesis of this disease is not fully understood, but is known to involve oxidative stress, deriving in part from mitochondrial dysfunction and inflammation (Hald and Lotharius, 2005). PPARγ agonists are reported to attenuate inflammatory responses in mouse models of PD, as measured by reductions in microgliosis and astrogliosis (Dehmer et al., 2004; Breidert et al., 2002; Schintu et al., 2009; Carta et al., 2011), and therefore the neuroprotective actions of PPARγ agonists are primarily considered to result from their anti-inflammatory actions. However, there is *in vitro* evidence to suggest that the protection of PPARγ agonists may also be due in part to modulation of the oxidative stress response (Jung et al., 2007). This study uses the 1-methyl-4-phenyl-1,2,3,6-tetrahydropyridine (MPTP) model of PD to further explore the role of anti-oxidant mechanisms in the neuroprotective actions of PPARγ agonists. It also seeks to address whether these effects are mediated by PPARγ as PPARγ agonists have been reported to have biological actions which do not require the activation of PPARγ (Chintharlapalli et al., 2005; Davies et al., 2001; Wang et al., 2011). MPTP is a neurotoxin that can penetrate the blood brain barrier where it is converted by monoamine oxidase-B in non-neuronal cells to its toxic metabolite 1-methyl-4-phenylpyridinium (MPP^+^) which is selectively taken up by dopaminergic cells of the nigrostriatal pathway (Jackson-Lewis and Przedborski, 2007). This toxin can be used *in vitro* in neuronal cultures as MPP^+^ and *in vivo* as MPTP.

## Experimental procedures

### Chemicals

Rosiglitazone and GW9662 were from Alexis Biochemicals (Exeter, UK). MPTP and MPP^+^ were from SigmaAldrich (Poole, UK). All other chemicals unless otherwise stated were of analytical grade.

### Cell culture

Human neuroblastoma SH-SY5Y cells were cultured in Dulbecco's Modified Eagle Medium (DMEM; SigmaAldrich) supplemented with 10% foetal calf serum (Biosera, Ringmer, East Sussex, UK) and 100 units/ml penicillin/streptomycin/glutamine (Invitrogen, Paisley, UK). Cells were kept at 37 °C in humidified 5% carbon dioxide and 95% air. Cells were seeded at 6000 cells/well in 96 well plates. All experiments were carried out 48 h after seeding and in serum-free media. Rosiglitazone and GW9662 were dissolved in dimethyl sulfoxide (DMSO) to make 1 mM solutions that were subsequently diluted with Dulbecco's phosphate buffered saline (DPBS; SigmaAldrich) and DMEM supplemented with 100 units/ml penicillin/streptomycin for experimental use. Final solutions contained 0.1% DMSO (v/v). MPP^+^ was dissolved in serum-free media and used at a final concentration of 1.5 μM. In experiments where rosiglitazone and GW9662 were used together with MPP^+^, cells were pre-treated with rosiglitazone or GW9662 for 16 h before the addition of MPP^+^. For co-treatment experiments cells were pre-treated with GW9662 for 16 h to ensure a high level of PPARγ inactivation and to allow exploration of the PPARγ dependence of the protective effects of rosiglitazone.

### Measurement of cell viability

Cell viability was determined by the conversion of the tetrazolium salt, 3-(4,5-dimethylthizol-2-yl)-2,5-diphenyltetrazolium bromide (MTT; Invitrogen) to its insoluble formazan. After treatments 10 μl of MTT solution (5 mg/ml) was added to the plated cells and incubated at 37 °C for 4 h. Media were then removed and the formazan solubilised in 100 μl DMSO. The absorption of the resulting solution was measured at 570 nm with reference at 670 nm using a PowerWave XS microplate spectrophotometer (Bio-Tek, Potton, Bedfordshire, UK).

### Measurement of lactate dehydrogenase release

Release of lactate dehydrogenase (LDH) into the culture media from cells with damaged membranes was measured using an assay kit (Cayman Chemicals, Ann Arbor, MI) as per manufacturer's instructions.

### Measurement of reactive oxygen species production

Reactive oxygen species (ROS) production was measured using the conversion of 2′,7′-dichlorofluorescein diacetate (DCF-DA; SigmaAldrich) to 2′,7′-dichlorofluorescein (DCF). Cells were plated and following treatments were rinsed with DPBS and incubated with 40 μM DCF-DA for 30 min at 37 °C. Cells were then rinsed in DPBS and lysed overnight in the dark in 100 μl RIPA buffer (50 mM Tris–HCl, pH 8; 150 mM NaCl; 1% NP-40; 0.5% sodium deoxycholate; 0.1% SDS and protease inhibitors (cOmplete Mini EDTA-free cocktail, Roche Diagnostics, Lewes, UK)) at 4 °C with constant agitation. Fifty microlitres of lysate was then taken for measurement of ROS production by comparison to a standard curve of DCF with absorbance read at 500 nm. Twenty five microlitres of lysate was taken for a bicinchoninic acid (BCA) assay (as per manufacturer's instructions (Pierce, Rockford, IL)) to allow ROS production to be normalised to protein levels. One millimolar l-buthionine-sulfoximine (BSO; SigmaAldrich) was used as a positive control.

### Measurement of superoxide dismutase and glutathione-S-transferase activity

Cells were plated in 25 cm^2^ flasks and following treatments were extracted in phosphate buffer (100 mM potassium phosphate, 2 mM EDTA pH 7.0). Superoxide dismutase activity was then assayed using an Epigentek (Brooklyn, NY) kit as per manufacturer's instructions. Glutathione-S-transferase activity was assessed using a Cayman Chemicals kit as per manufacturer's instructions.

### Animals and drug treatments

All mice were housed individually with a 12-hour/12-hour light/dark cycle with free access to food and water. All procedures were in accordance with the Animals (Scientific Procedures) Act 1986 and were approved by the Home Office, Dundee, UK. MPTP handling and safety measures were consistent with Jackson-Lewis and Przedborski (2007). Twelve week-old male C57BL6 mice (Charles River Laboratories, UK) (3–7 mice per timepoint) received intraperitoneal injections of MPTP-HCl (30 mg/kg free base — SigmaAldrich, Poole, UK) dissolved in saline, one injection for five consecutive days, and were killed at selected times ranging from 0 to 21 days after the last injection. Control mice received saline only. For treatment with PPARγ agonist or antagonist twelve week-old male C57BL6 mice received intraperitoneal injections of 10 mg/kg rosiglitazone, 5 mg/kg of GW9662, or vehicle (5% DMSO v/v) daily for 3 days prior to MPTP administration, throughout MPTP administration (30 mg/kg for five consecutive days) and for 21 days following MPTP administration. Mice were sacrificed 21 days after last MPTP injection.

### PPARγ, tyrosine hydroxylase (TH), glial fibrillary acid protein (GFAP), ionized calcium-binding adaptor molecule 1 (Iba1) and NeuN immunohistochemistry

This was performed as described (Teismann et al., 2003) with the minor modification that incubation with primary antibodies was performed sequentially as detailed below. Firstly incubation with either rabbit anti-PPARγ (1:100; Alexis, San Diego, CA) or mouse anti-PPARγ depending on host species of dual staining antibody, overnight at 4 °C. Then 3 × 5 min washes in phosphate-buffered saline solution (PBS) containing 0.1% Triton followed by incubation with mouse anti-NeuN (1:100 Chemicon, Temecula, CA), mouse or rabbit anti-tyrosine hydroxylase (TH; 1:500; Chemicon), mouse anti-human glial fibrillary acidic protein (GFAP; 1:100; DAKO, Cambridgeshire, UK) or rabbit anti-ionized calcium-binding adaptor molecule 1 (Iba1; 1:1000; Wako Chemicals, Neuss, Germany) overnight at 4 °C. Immunostaining was visualised with Alexa Fluor 488 anti-rabbit or anti-mouse (1:300; Molecular Probes, Eugene, OR) and cy-3 anti-rabbit or anti-mouse (1:200; Jackson Immuno Research, West Grove, PA). Immunostaining was visualised by confocal microscopy (LSM 510, Carl Zeiss, Hertfordshire, UK).

### RNA extraction and quantitative RT-PCR

Total RNA was extracted from selected brain regions using the TRIzol (Invitrogen) homogenization method as in the manufacturer's instructions. Samples were then subjected to a DNase digestion, DNase I Amp Grade kit (Invitrogen), and first strand cDNA synthesis was carried out using the Superscript II kit (Invitrogen). The primer sequences used in this study are detailed in Table 1. Quantitative PCR amplification was undertaken using the Lightcycler 480 and the Lightcycler 480 SYBR green I Master (Roche Diagnostics, Lewes, UK) as in the manufacturer's guidelines with annealing temperatures as detailed in Table 1. The identity of fragments amplified with these primers was confirmed by DNA sequencing performed by The Sequencing Service (College of Life Sciences, University of Dundee, Scotland, www.dnaseq.co.uk) using Applied Biosystems Big-Dye Ver 3.1 chemistry on an Applied Biosystems model 3730 automated capillary DNA sequencer.

### Western blot analysis

Total proteins from mouse ventral midbrain, striatum and cerebellum samples were isolated in NP-40 buffer (20 mM Tris–HCl pH 8; 137 mM NaCl; 10% glycerol; 1% NP-40; 2 mM EDTA and protease inhibitors (cOmplete Mini EDTA-free cocktail, Roche)) 1:20 (wt/vol). Total proteins from human post-mortem ventral midbrain, and striatum were isolated in NP-40 buffer 1:5 (wt/vol). Protein concentration was determined using a BCA kit. After boiling in Laemmli's buffer, 20 μg of protein was separated by electrophoresis on a 10% sodium dodecyl sulphate–polyacrylamide gel, transferred to nitrocellulose membrane, and blocked with 5% non-fat dried milk in PBS containing 0.05% Tween-20 (vol/vol) for 1 h. Incubation with rabbit anti-PPARγ (1:1000; Alexis), rabbit anti-caspase 3 (1:2500; Abcam), rabbit anti-superoxide dismutase 1 (1:1000; Assay Designs, Ann Arbor MI) rabbit anti-glutathione-S-transferase π (1:1500; Assay Designs) or mouse anti-β-actin (1:25,000; SigmaAldrich) overnight at 4 °C followed. Blots were then washed in PBS-Tween (0.05%) and incubated with either an anti-rabbit (1:5000) or anti-mouse (1:10,000) conjugated horseradish peroxidase antibody (Amersham Biosciences, Buckinghamshire, UK) at room temperature for 1 h. Blots were then washed in PBS-Tween (0.05%) and developed using a chemiluminescence solution (1 ml (50 mg luminol sodium salt (SigmaAldrich) in 200 ml 0.1 M Tris–HCl pH 8.6), 100 μl (11 mg *p*-coumaric acid (SigmaAldrich) in 10 ml DMSO) and 0.3 μl 30% hydrogen peroxide). Bands were visualised with an AlphaInnotech digital imaging system (San Leandro, CA) and quantified with AlphaEase FC 5.02 software.

### Stereological counting and analysis of striatal TH immunoreactivity

Immunostaining for stereological counting of TH and Nissl-stained SNpc neurons was carried out on midbrain sections as described in Teismann et al. (2003). Every 4th section was taken until there were 12 sections for each substantia nigra pars compacta (SNpc). The primary antibody was a polyclonal rabbit anti-TH (1:1000; Chemicon) and staining was visualised with 3,3′-diaminobenzidine (SigmaAldrich). The sections were counted using light microscopy (AxioImager M1, Carl Zeiss) and the optical fractionator method (West, 1993) (Stereo Investigator version 7, MBF Bioscience, Magdeburg, Germany).

For analysis of striatal TH-immunoreactivity, every 8th section of the striatum stained as described (Teismann et al., 2003) (rabbit anti-TH (1:500; Chemicon)). TH immunoreactivity was assessed on scans (Hewlett Packard Scanjet G3110, Bracknell, Berkshire, UK) of the sections using Scion Image (Version 4.0.3.2 Scion Corporation, MD).

### HPLC analysis of striatal dopamine and 3,4-dihydroxyphenylacetic acid (DOPAC) levels

High-performance liquid chromatography (HPLC) with electrochemical detection was used to measure striatal levels of dopamine, and DOPAC using a method that has been described (Nuber et al., 2008). Briefly, mice were killed, 21 days after the last MPTP injection, and the striata were dissected out and snap frozen on solid carbon dioxide. Striata were then homogenised in 0.1 M perchloric acid (1:30 wt/vol), sonicated and centrifuged at 18,600 ×*g* at 4 °C for 20 min. Following centrifugation 20 μl of sample was injected onto a C18 column (Dionex, Germering, Germany). The mobile phase consisted of 90% 50 mM sodium acetate, 35 mM citric acid, 105 mg/l octane sulfonic acid, 48 mg/l sodium EDTA solution, and 10% methanol at pH 4.3. Flow rate was 1 ml/min. Peaks were detected by an ESA Coulchem II electrochemical detector (ESA, Dionex), and the detector potential was set at 700 mV. Data were collected and processed using the Chromeleon computer system (Dionex).

### Measurement of striatal MPP^+^ levels

Liquid chromatography with on-line ultraviolet detection/tandem mass spectrometry (LC-UV-MS-MS) was used to measure striatal levels of MPP^+^. Briefly, mice received drug treatment as outlined in Animals and drug treatments section and 90 min after a single MPTP injection (25 mg/kg) mice were sacrificed. The striata were dissected out and snap frozen on solid carbon dioxide. Striata were then sonicated in 0.1 M perchloric acid (1:30 wt/vol), and centrifuged at 14,000 rpm (18,620 ×*g*; Mikro 200R) at 4 °C for 20 min. Following centrifugation 2 μl of sample was injected onto a Hichrom 5μ C18 column (Hichrom, Theale, UK). The mobile phase consisted of 80% 0.1% formic acid in water/20% 0.1% formic acid in acetonitrile. Flow rate was 200 μl/min. MPP^+^ was detected by a photodiode array detector set at 295 nm, and a triple quadrupole mass spectrometry with a mass to charge ratio of 170–128 at 32 V and 1.9 mTorr (ThermoSurveyor PDA/TSQ Quantum, ThermoScientific, Loughborough, UK). Data were collected and processed using Xcalibur 2.0.7 SP1.

### Statistical analysis

Data was analysed in SigmaPlot 11 for Windows (Systat Software Inc., Chicago, IL). All values are expressed as the mean ± SEM. Normal distribution of the data was tested and the homogeneity of variance confirmed with Levene test. For single pairs of data Student t-tests were used for comparisons between means. For data sets greater than single pairs ANOVA was used to analyse differences among means with time or treatment as the independent factor. When ANOVA showed significant differences post hoc testing was used to make comparisons between means, Dunnett's post hoc test was used for time-course studies and Student Newman–Keuls was used to make pairwise comparisons in all other studies. Data not normally distributed was analysed with the Kruskal–Wallis test followed by Mann Whitney U-tests. The null hypothesis was rejected at the 0.05 level.

## Results

### Impacts of a PPARγ agonist and antagonist on MPP^+^-induced cytotoxicity *in vitro*

**Fig. 1:**
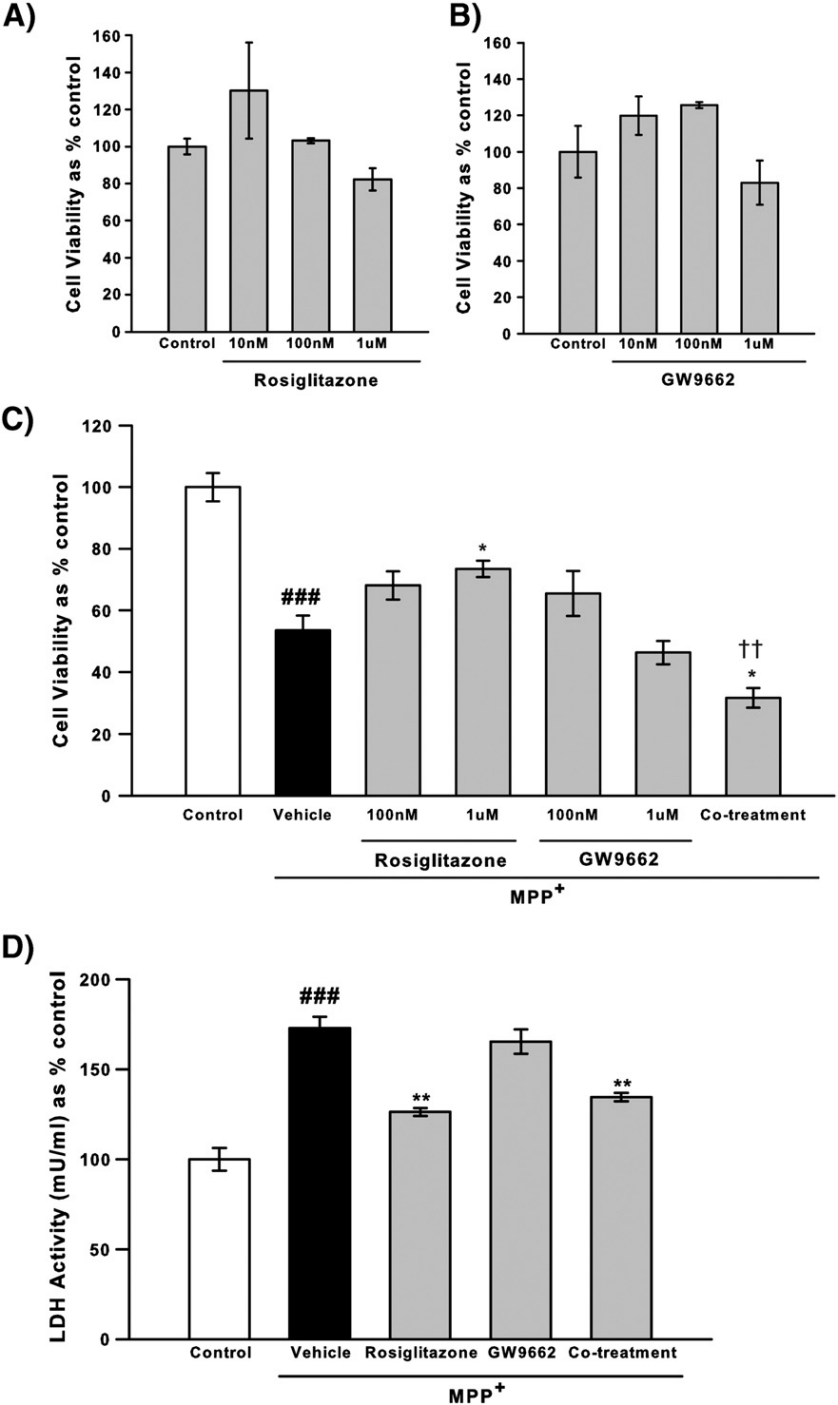
Effects of the PPARγ agonist, rosiglitazone and the antagonist, GW9662 on MPP^+^ cytotoxicity as measured by cell viability and lactate dehydrogenase (LDH) release. The impact of rosiglitazone (A) and GW9662 (B) on cell viability was assessed by MTT reduction, no alterations in cell viability were seen. After 48 h MPP^+^ (1.5 mM) decreased cell viability in vehicle (0.1% DMSO) treated cells, this was attenuated by rosiglitazone (1 μM) (C). Co-treatment with GW9662 (1 μM) prevented the protective effects of rosiglitazone. LDH release was increased after MPP^+^ treatment (1.5 mM) and again this was attenuated by rosiglitazone (1 μM) (D). However, GW9662 (1 μM) did not prevent the protective effects of rosiglitazone in this assay. Data are mean ± SEM, n = 3, ###*p* < 0.001 MPP^+^ compared to control; *p < 0.05; **p < 0.01 compared to MPP^+^ with vehicle, ††*p* < 0.01 co-treatment compared to rosiglitazone treatment (ANOVA followed by Student Newman–Keuls post hoc test).

Neither the PPARγ agonist rosiglitazone nor the antagonist GW9662 alone had any impacts on cell viability as measured by assessment of metabolic status by the reduction of MTT (Figs. 1A and B). Cell viability was reduced by 56.5% by MPP^+^ treatment compared to vehicle alone (*p* < 0.001 ANOVA, Student Newman–Keuls post hoc test; Fig. 1C). Next to assess the impact of rosiglitazone and/or GW9662 on MPP^+^-induced cytotoxicity an initial pretreatment strategy which then continued throughout MPP^+^ treatment was used. This strategy was utilised as to accurately reflect the *in vivo* dosing regime (see Animals and drug treatments and Effects of PPARγ antagonist GW9662 on MPTP toxicity). This decrease was attenuated by treatment with rosiglitazone at both 100 nM and 1 μM although only the latter reached statistical significance (*p* = 0.078 100 nM; *p* < 0.05 1 μM ANOVA, Student Newman–Keuls post hoc test). Treatment with GW9662 at 1 μM did not impact on MPP^+^-induced toxicity. However co-treatment with rosiglitazone (1 μM) and GW9662 (1 μM) (see Cell culture section for details) prevented the protective effect of rosiglitazone alone (*p* < 0.01 ANOVA, Student Newman–Keuls post hoc test) suggesting not simply a PPARγ independent mechanism, but rather having an additive or potentiating toxic effect.

A second measure of cell viability that does not involve metabolic status, the LDH assay which assesses membrane integrity, was used as PPARγ has many roles in cell metabolism (Desvergne and Wahli, 1999). In this assay MPP^+^ increased LDH activity (*p* < 0.001 ANOVA, Student Newman–Keuls post hoc test; Fig. 1D) and again this was attenuated by rosiglitazone treatment (*p* < 0.01 ANOVA, Student Newman–Keuls post hoc test) and unaffected by GW9662 treatment. In contrast to the cell viability assay co-treatment did not prevent the protective effect of rosiglitazone, suggesting that the impacts of rosiglitazone on this measure of cytotoxicity do not require PPARγ-activation.

### Impacts of a PPARγ agonist and antagonist on MPP^+^-induced oxidative stress *in vitro*

**Fig. 2:**
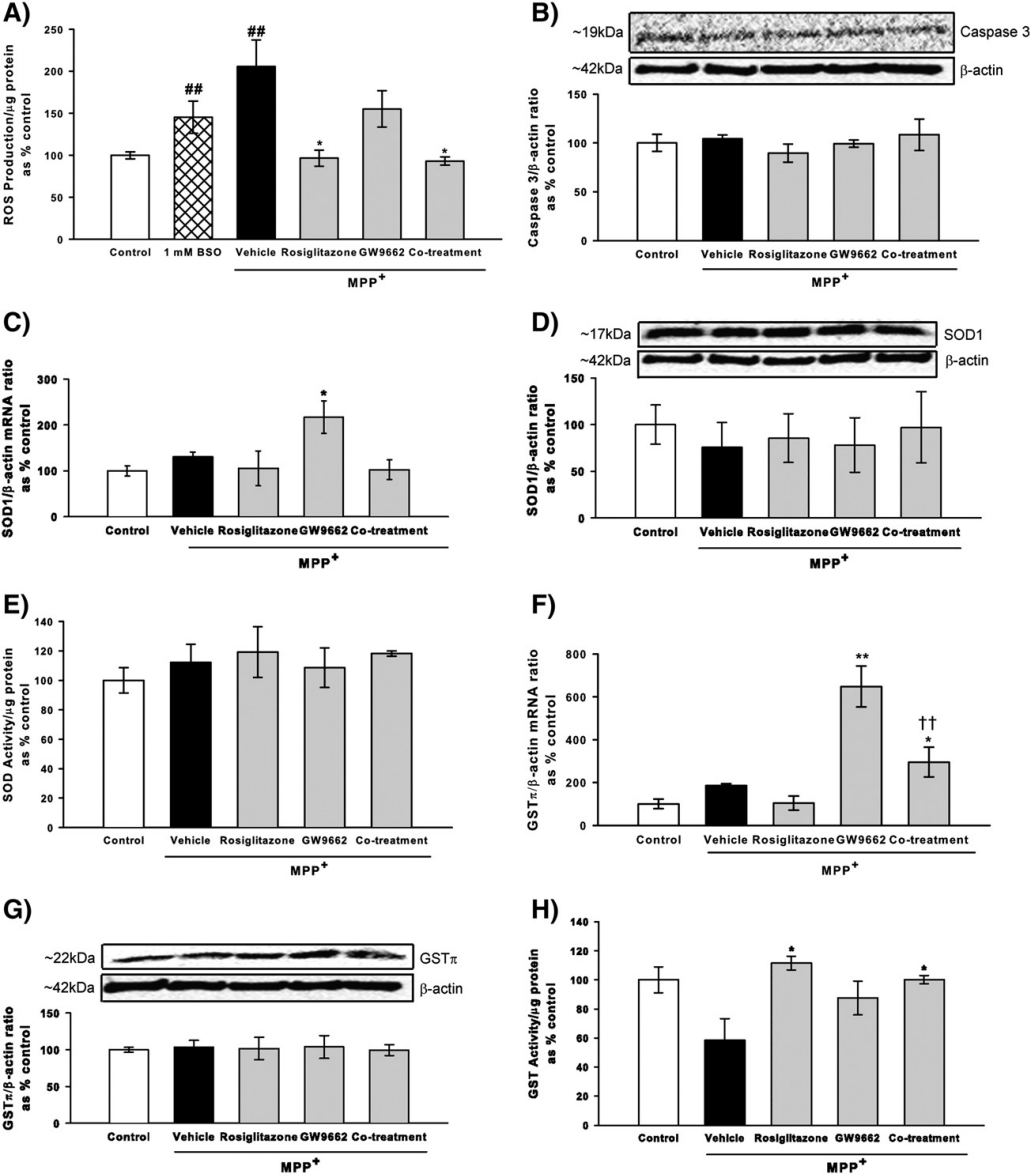
Effects of the PPARγ agonist, rosiglitazone and the antagonist, GW9662 on MPP^+^-induced reactive oxygen species (ROS) formation and alterations in the expression of caspase 3 and anti-oxidant enzymes. ROS formation was assessed by conversion of DCF-DA to DCF. After 24 h both MPP^+^ (1.5 mM) and l-buthionine-sulfoximine (1 mM, BSO) had induced ROS formation (A). Rosiglitazone (1 μM) attenuated the MPP^+^-induced ROS formation and this protective effect was unaltered by co-treatment with GW9662 (1 μM). Levels of activated caspase 3 protein were assessed 24 h after MPP^+^ treatment and shown no change (B). Levels of superoxide dismutase and glutathione-S-transferase-π (GSTπ) mRNA and protein after treatment with MPP^+^, rosiglitazone, GW9662 or co-treatment of rosiglitazone and GW9662 were evaluated. Only treatment with GW9662 increased SOD1 and GSTπ mRNA (C and F), and these changes did not correlate with changes in protein levels (D and G). No alteration in SOD activity was seen (E). MPP^+^ reduced GST activity non-significantly and this reduction was attenuated by both rosiglitazone and co-treatment (H). Data are mean ± SEM, n = 3, ##*p* < 0.01 MPP^+^ compared to control; *p < 0.05; **p < 0.01 compared to MPP^+^ alone, ††*p* < 0.01 co-treatment compared to rosiglitazone treatment (ANOVA followed by Student Newman–Keuls post hoc test; Kruskal–Wallis test followed by Mann Whitney-U post hoc test) for ROS formation and GST activity; ROS — reactive oxygen species; SOD1 — superoxide dismutase 1; GST — Glutathione-S-transferase.

To further explore the protective mechanisms of rosiglitazone and the PPARγ dependence of these effects the generation of reactive oxygen species (ROS) was assessed. l-Buthionine-sulfoximine (BSO) was used as a positive control to ensure ROS generation was detectable and after 24 h of treatment with 1 mM BSO ROS levels were 1.45-fold higher than those of the control (*p* < 0.01 Kruskal–Wallis test, Mann Whitney-U post hoc test). Twenty-four hours of MPP^+^ treatment induced ROS levels over 2-fold higher than those in control cells (*p* < 0.01 Kruskal–Wallis test, Mann Whitney-U post hoc test; Fig. 2A). This increase was attenuated by the rosiglitazone treatment (*p* < 0.05 Kruskal–Wallis test, Mann Whitney-U post hoc test) back to the level of control cells. GW9662 did not affect MPP^+^-induced ROS formation. Co-treatment with GW9662 did not affect the protective effects of rosiglitazone treatment suggesting that the protective effect of rosiglitazone does not require PPARγ activation (*p* < 0.05 Co-treatment *vs*. MPP^+^ alone Kruskal–Wallis test, Mann Whitney-U post hoc test).

These data suggest that treatment with rosiglitazone attenuates MPP^+^-induced ROS formation without PPARγ activation, to confirm this the role of two anti-oxidant genes implicated in MPTP toxicity and known to be PPARγ responsive – Cu/Zn superoxide dismutase (SOD1) (Yoo et al., 1999; Fornai et al., 2002) and GSTπ (Park et al., 2004; Smeyne et al., 2007) was investigated 24 h after MPP^+^ treatment. This timepoint was chosen as ROS formation was significantly increased, but alterations in caspase 3 levels indicative of cell death were not detected (Fig. 2B). Only treatment with GW9662 increased SOD1 mRNA levels when compared to MPP^+^ treated cells (*p* < 0.05 ANOVA, Student Newman–Keuls post hoc test; Fig. 2C). This did not correlate to an increase in SOD1 protein levels (Fig. 2D). The lack of effect of rosiglitazone on SOD1 was further confirmed as no alterations in SOD activity (Cu/Zn and Mn SOD activity were assessed together) were seen when compared to control cells 24 h after MPP^+^ administration (Fig. 2E). Expression of GSTπ mRNA was increased with GW9662 and co-treatment (GW9662 *vs*. MPP^+^ *p* < 0.01; co-treatment *vs*. MPP^+^ *p* < 0.05 ANOVA, Student Newman–Keuls post hoc test; Fig. 2F). Similar to SOD1 these increases were not reflected at the protein level (Fig. 2G). However alterations in global GST activity (all GST isoforms, including GSTπ) were detected (Fig. 2H), treatment with MPP^+^ demonstrated a trend towards reduced GST activity (*p* = 0.073 compared to control cells Kruskal–Wallis test, Mann Whitney-U post hoc test). This decrease was attenuated by both rosiglitazone and co-treatment (*p* < 0.05 rosiglitazone *vs*. vehicle; *p* < 0.05 co-treatment *vs*. vehicle; Kruskal–Wallis test, Mann Whitney-U post hoc test) and unaffected by treatment with GW9662, supporting the independence of these effects from PPARγ activation.

### Effects of MPTP treatment on PPARγ levels *in vivo*

**Fig. 3:**
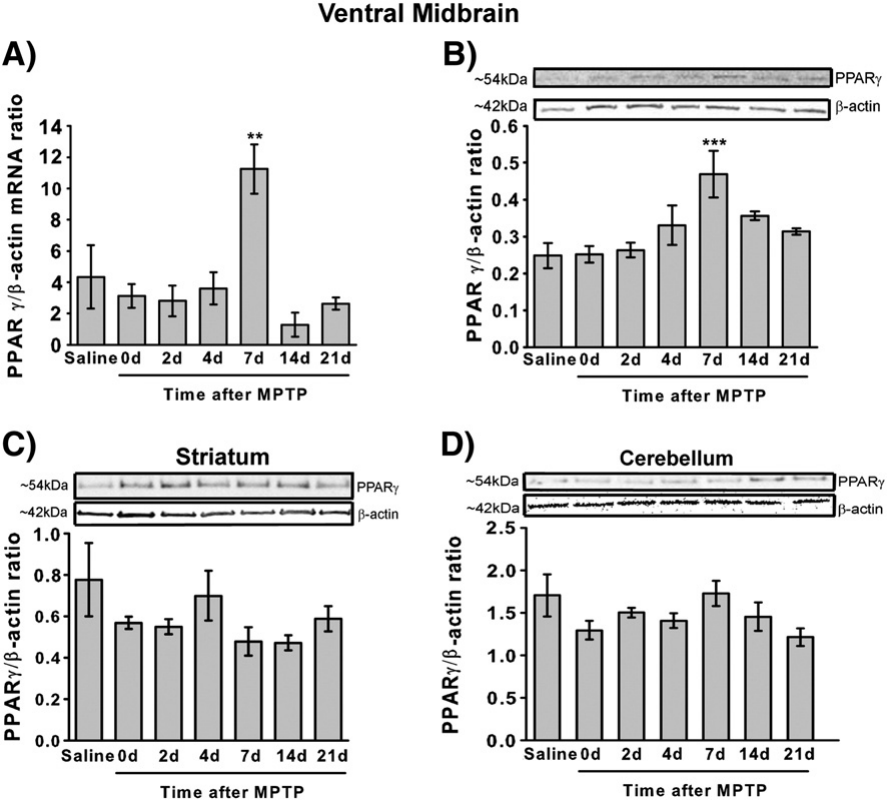
Alterations in PPARγ expression following MPTP treatment. PPARγ mRNA levels in the ventral midbrain are increased seven days after MPTP compared to saline treated mice (A), and this corresponds to an increase in PPARγ protein levels (B). PPARγ protein levels are unchanged in the striatum (C) and cerebellum (D). Data are mean ± SEM, n = 3–6 mice per timepoint. **p < 0.01, ***p < 0.001 compared to saline (ANOVA with student Newman–Keuls post hoc test) (d — days after MPTP (5 × 30 mg/kg daily) administration).

As demonstrated *in vitro* the PPARγ agonist rosiglitazone has anti-oxidant effects that appear not to require PPARγ activation, and which may contribute to the previously documented neuroprotective effects of rosiglitazone in *in vivo* models of PD (Schintu et al., 2009; Carta et al., 2011). In order to determine if the *in vitro* anti-oxidant effects of rosiglitazone could be translated to an *in vivo* model of PD, first any alterations in PPARγ levels in the substantia nigra pars compacta (SNpc), the main area of neuron loss in PD, were examined by western blot and qPCR analysis. Quantitative PCR showed a significant increase in PPARγ mRNA levels in the ventral midbrain (the area containing the SNpc) 7 days after MPTP administration compared to saline-treated mice (*p* < 0.01 ANOVA, Student Newman–Keuls post hoc test; Fig. 3A) when normalised to β-actin levels (β-actin levels were unchanged by MPTP treatment, data not shown). This increase correlated with a rise in PPARγ protein levels (*p* < 0.001 ANOVA, Student Newman–Keuls post hoc test; Fig. 3B). No alterations in PPARγ protein levels were seen in either the striatum (Fig. 3C) or the cerebellum (Fig. 3D).

**Fig. 4:**
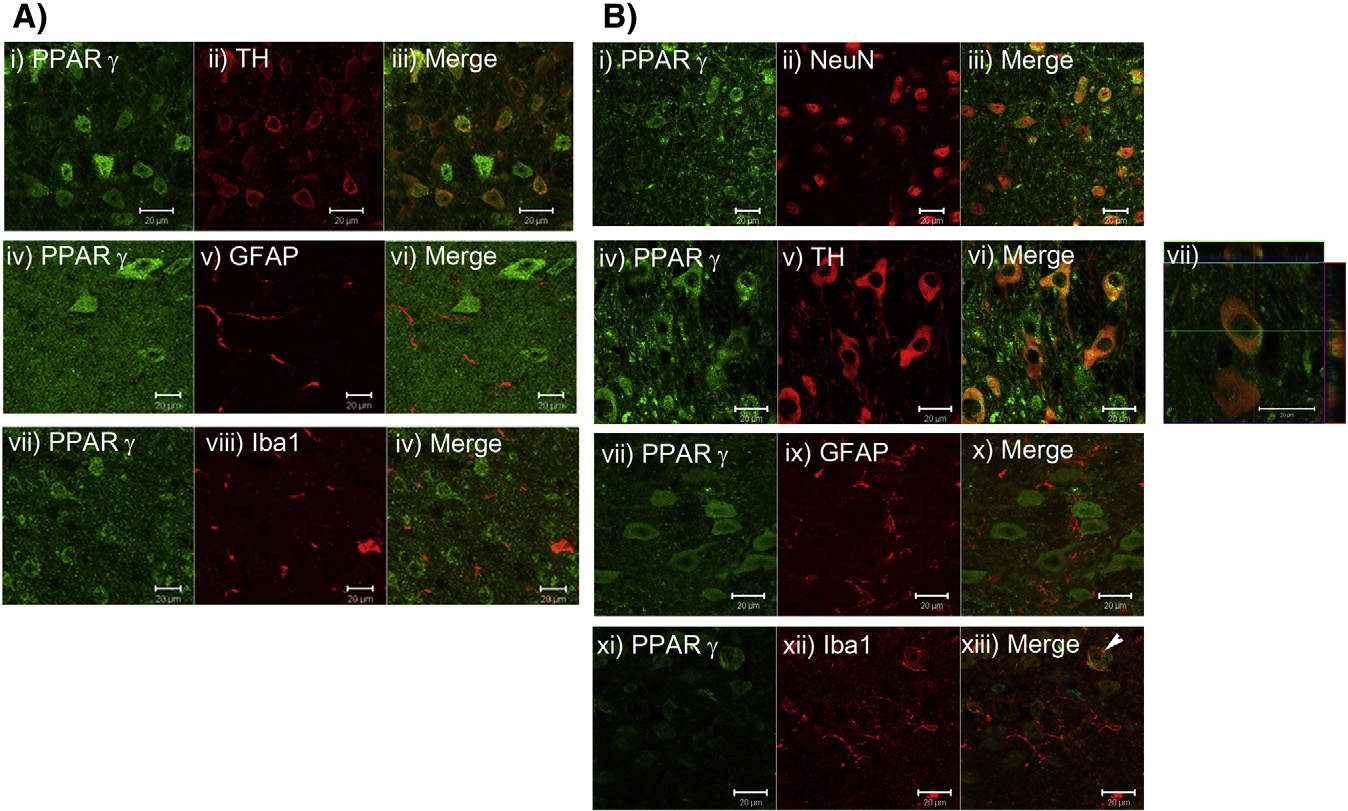
PPARγ immunolocalisation in saline and MPTP treated mice. Double immunofluorescence confirms that PPARγ (green) is expressed in neuronal nuclei in the substantia nigra pars compacta with NeuN (A and B i–iii; red), including in TH-positive neurons (A and B iv–vi and vii; red), in both saline and MPTP treated mice. PPARγ did not colocalise with GFAP-positive astrocytes (A and B viii–x; red) in either saline or MPTP treated mice. Colocalisation of PPARγ with Iba1-positive microglia was seen seven days after MPTP treatment (B xi–xiii; red). (Scale bars are 20 μm).

Subsequently the cellular localisation of PPARγ in the SNpc was ascertained in both saline- and MPTP-treated mice to determine if a particular cell type was responsible for the upregulation in PPARγ levels seen after MPTP treatment. PPARγ is expressed in a peri-nuclear location in neurons, including dopaminergic TH-positive cells in the SNpc (Fig. 4A i–iii, and Fig. 4B i–iii, iv–vi and vii) in both saline- and MPTP-treated mice. No expression of PPARγ was detected in astrocytes of either saline or MPTP treated mice, nor microglia of saline treated mice (Fig. 4A iv–vi, vii–ix and Fig. 4B vii–x). However, PPARγ expression was detected in amoeboid microglial cells 7 days after MPTP treatment (Fig. 4B xi–xii), suggesting that this cell type may be the cell type responsible for the upregulation of PPARγ levels seen with MPTP treatment.

### Effects of PPARγ antagonist GW9662 on MPTP toxicity

**Fig. 5:**
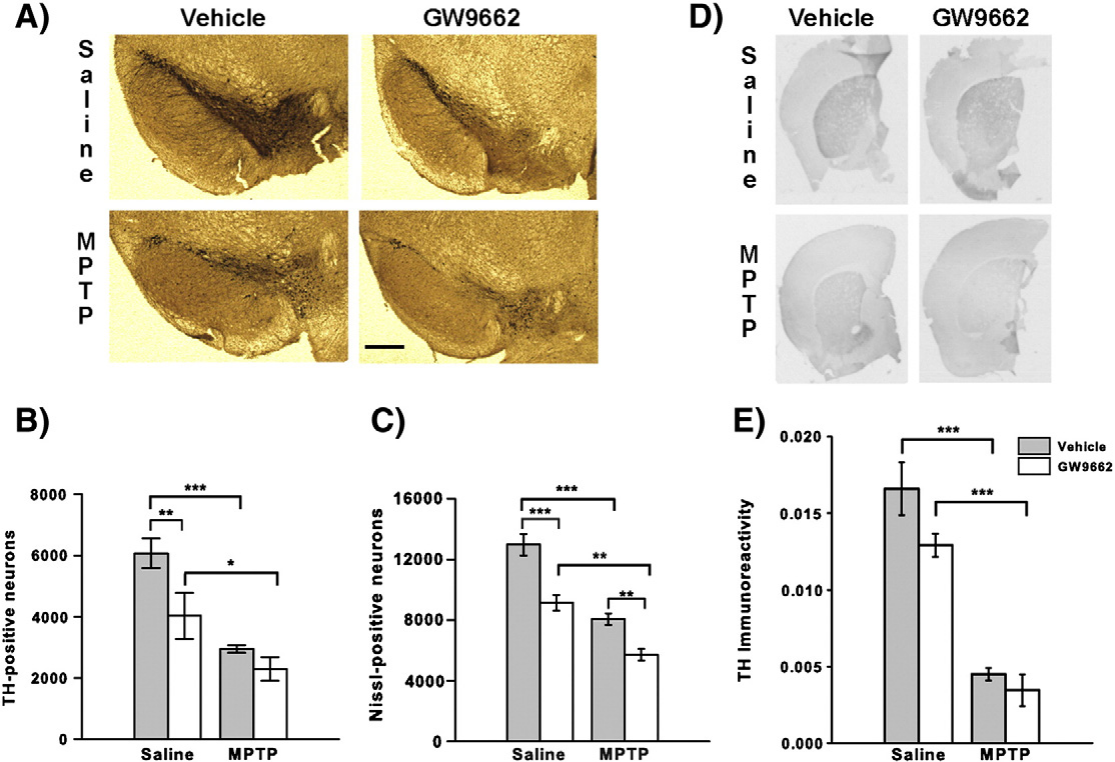
Effects of GW9662 treatment on MPTP neurotoxicity. GW9662 treatment alters neuronal sensitivity to MPTP toxicity. (A) Representative micrographs of TH and Nissl stained micrographs. Both TH-positive neuron (B) and Nissl neuron (C) numbers were reduced by GW9662 treatment in saline-treated mice compared to vehicle treated mice (5% DMSO). MPTP (5 × 30 mg/kg daily i.p.) reduced TH-positive and Nissl-positive neuron number. GW9662 treatment enhanced the impact of MPTP on Nissl-positive neurons only. No differences were detected in striatal TH-immunoreactivity (D and E) between mice treated with GW9662 and vehicle treated mice. Data are mean ± SEM, n = 4–5 mice per group. *p < 0.05; **p < 0.01; ***p < 0.001 (ANOVA with Student Newman–Keuls post hoc test).

The protective effect of rosiglitazone and other glitazones against MPTP toxicity has been reported elsewhere (Schintu et al., 2009; Dehmer et al., 2004; Breidert et al., 2002). To permit the work of this study to be comparable with these studies a pretreatment strategy as outlined in Animals and drug treatments section was adopted. However, in this study rosiglitazone significantly reduced striatal MPP^+^ levels when compared to vehicle (5% DMSO) (*p* < 0.05 Kruskal–Wallis test, Mann Whitney U post hoc test; Table 2) thus indicating that it was interfering with MPTP bioactivation preventing its use in this MPTP model as it would not be possible to definitively attribute any protective effects seen to rosiglitazone itself. Subsequently it was necessary to address the role of PPARγ in dopaminergic neuron death following MPTP treatment using an inhibitory approach. PPARγ inhibition was tested by the administration of the PPARγ antagonist GW9662 as PPARγ knock-out mice are embryonically lethal (Barak et al., 1999) and conditional knock-out mice were unavailable. MPTP treatment reduced both TH- and Nissl-positive neurons in the SNpc compared to saline-treated mice in both treatment groups (TH 6065 ± 485 *vs*. 2948 ± 128, *p* < 0.001 for vehicle and 4030 ± 759 *vs*. 2295 ± 395, *p* < 0.05 for GW9662 treatment; Nissl 12960 ± 698 *vs*. 8056 ± 319, *p* < 0.0001 for vehicle and 9150 ± 514 *vs*. 5725 ± 373, *p* < 0.01 for GW9662 treatment). GW9662 alone reduced TH and Nissl neuron numbers in saline-treated mice (*p* < 0.01 TH and *p* < 0.001 Nissl ANOVA, Student Newman–Keuls post hoc test; Figs. 5A–C). In combination with MPTP only Nissl neuron numbers were further reduced by GW9662 treatment compared to MPTP and vehicle (*p* = 0.006 ANOVA, Student Newman–Keuls post hoc test).

The effect of GW9662 on MPTP-induced striatal damage was evaluated by measuring TH immunoreactivity and HPLC measurement of the levels of dopamine and its metabolite DOPAC. MPTP induced a reduction in striatal TH immunoreactivity in both vehicle- and GW9662-treated mice (*p* < 0.001 for both groups ANOVA, Student Newman–Keuls post hoc test; Figs. 5D and E). This reduction was not affected by GW9662 treatment, however, GW9662 did show a trend towards reduction of striatal TH immunoreactivity in saline-treated mice (*p* = 0.052 ANOVA, Student Newman–Keuls post hoc test). MPTP administration induced significant reductions in the levels of dopamine and DOPAC in the striatum detected by HPLC in both groups (Dopamine *p* < 0.05 vehicle and *p* < 0.001 GW9662; DOPAC *p* < 0.01 vehicle and *p* < 0.001 GW9662 Kruskal–Wallis test, Mann Whitney U post hoc test; Table 3). GW9662 treatment did not affect dopamine and DOPAC levels in saline-treated mice.

## Discussion

PPARγ agonists have been shown to be neuroprotective in a number of neurodegenerative diseases (Escribano et al., 2009; Kiaei et al., 2005; Feinstein et al., 2002; Niino et al., 2001) including PD (Dehmer et al., 2004; Breidert et al., 2002; Schintu et al., 2009; Carta et al., 2011). These effects are predominantly considered to result from the anti-inflammatory actions mediated by PPARγ activation (Dehmer et al., 2004; Schintu et al., 2009; Kiaei et al., 2005), indeed both rosiglitazone and pioglitazone have been shown to reduce microgliosis and astrogliosis in the MPTP mouse model of PD (Dehmer et al., 2004; Breidert et al., 2002; Schintu et al., 2009). There is also growing evidence that PPARγ agonists have anti-oxidant effects (Nicolakakis et al., 2008; Xiong et al., 2008; Jung et al., 2007) and PD pathogenesis also involves oxidative stress (Jenner, 2003). In this study administration of rosiglitazone attenuated MPP^+^-induced alterations in SH-SY5Y cell viability which was coincident with a reduction in MPP^+^-induced ROS production. The use of the PPARγ antagonist, GW9662, suggests that these effects do not require PPARγ activation as in the majority of experiments in this study co-treatment did not attenuate the protective effects of rosiglitazone. The exception to this was the MTT assay measuring cell viability, where co-treatment with GW9662 and rosiglitazone did attenuate the protective effects of rosiglitazone alone, suggesting that the observed effect is due to an additive or potentiating toxic effect of the compounds. The independence of the effects seen on ROS formation from PPARγ activation is supported by the lack of change in the activity of SOD *in vitro*, as SOD1 is a key anti-oxidant enzyme with a known peroxisome proliferator response element (PPRE) (Yoo et al., 1999) and would be expected to change if the effects of rosiglitazone were PPARγ dependent. This lack of change in SOD activity is in contrast to previous work (Jung et al., 2007), but a different dose regime and timepoint were examined in that study. As the initial impact of rosiglitazone on oxidative stress was not mediated by increased SOD activity, it may have arisen from changes in GST activity as GSTs are important for detoxification of a number of oxidative stress products such as peroxidated polyunsaturated fatty acids and 4-hydroxy-2-alkenals (Hayes et al., 2005). Rosiglitazone did attenuate the MPP^+^-induced reduction in GST activity suggesting that modulation of GST activity is involved in the early anti-oxidant effects of rosiglitazone. Co-treatment of rosiglitazone and GW9662 also had a positive effect on GST activity suggesting that this protective effect of rosiglitazone may also not require PPARγ activation. PPARγ agonists have been documented to have actions that do not require PPARγ activation in previous studies (Chintharlapalli et al., 2005; Davies et al., 2001; Wang et al., 2011), including in a model of brain trauma where pioglitazone reduced the inflammatory response in a manner unaltered by the administration of the PPARγ antagonist T0070907 (Thal et al., 2011).

The *in vitro* work of this study shows rosiglitazone to have anti-oxidant effects that do not require PPARγ activation, and previous studies have shown rosiglitazone to have protective effects *in vivo* (Schintu et al., 2009; Carta et al., 2011) which may involve anti-oxidant effects in addition to the documented attenuation of microglial and astrocytic activation. Subsequently it was important to determine the relevance of our findings to the *in vivo* situation using the MPTP model of PD. PPARγ was expressed in the neurons of the SNpc correlating with previous work (Carta et al., 2011; Ou et al., 2006). PPARγ has also been documented to be expressed in microglia (Carta et al., 2011; Bernardo et al., 2000; Storer et al., 2005), and this was the case in MPTP-treated mice in this study, however, only Iba1-positive microglia of an amoeboid phenotype were positive for PPARγ. This is also consistent with Carta et al. (2011) who have shown that PPARγ co-localisation with a microglial marker, CD11b, is increased by MPTP treatment. As PPARγ levels were not upregulated until seven days after MPTP treatment it is unlikely that the PPARγ expression that occurs in activated microglia plays a significant role in MPTP toxicity, as the majority of dopaminergic neuron loss has occurred by this timepoint (Jackson-Lewis et al., 1995). However, the presence of PPARγ in activated microglia may underlie some of the positive effects of PPARγ agonists as these pharmacological interventions increase the negative regulatory effects of PPARγ on inflammation (Jiang et al., 1998; Ricote et al., 1998) and the reduction of microglia inflammatory responses has been shown to attenuate MPTP toxicity (Wu et al., 2002). The strong presence of PPARγ in neurons suggests that modulation of inflammatory responses may not be the whole picture.

Further investigations of the protective effects of rosiglitazone against MPTP were indicated however, rosiglitazone treatment was found to interfere with the metabolic activation of MPTP. This is in contrast to the work of Schintu et al. (2009) who did not see alterations in MPTP bioactivation following rosiglitazone treatment. However, the use of a clearance inhibitor, probenecid, and differences in the methodology used for measurement of MPP^+^ level means definitive comparisons between this study and that of Schintu et al. (2009) are not possible. Another glitazone, pioglitazone, has been shown to interfere with the activation of MPTP by inhibiting monoamine oxidase B (MAO-B), which converts MPTP to its active metabolite MPP^+^ (Quinn et al., 2008) suggesting that the glitazones as a drug class may be neuroprotective in the MPTP mouse model by a mechanism that is irrelevant to PD pathogenesis.

Consequently these data meant an inhibitory approach was necessary to address the role of PPARγ in the MPTP model of PD. Ideally a PPARγ null mouse would have been utilised, however, PPARγ null mice are not viable (Barak et al., 1999) and conditional PPARγ null mice were unavailable. Subsequently administration of GW9662, an irreversible PPARγ antagonist, was used. GW9662 was toxic to neurons, including dopaminergic neurons, in the SNpc of both saline and MPTP treated mice. As this study focussed on the brain regions affected in PD, namely the SNpc and the striatum, no other brain regions were assessed. It would, however, be interesting to further explore if treatment with GW9662 and subsequently PPARγ antagonism has global neurotoxicity as suggested by studies in other neurodegenerative diseases including Huntington's disease (Quintanilla et al., 2008) and Alzheimer's disease (Du et al., 2009).

Other PPARγ antagonists are implicated in neurotoxicity, bisphenol A diglycidyl ether (BADGE) worsens clinical scores in the experimental allergic encephalomyelitis model of multiple sclerosis (Raikwar et al., 2005). Taken together these data suggest that PPARγ activity is important for neuronal survival especially under conditions of cellular stress and this idea is further supported by increased susceptibility to cerebral ischemia in mice with a neuronal deficiency of PPARγ (Zhao et al., 2009). Further work is required to determine if the toxic impacts of GW9662 seen in the study require the presence of PPARγ. The mechanism of toxicity also needs further elucidation, to identify the novel pathway involved and whether these effects do not require PPARγ to be present or to ascertain which pathways involving PPARγ are important for these effects. One possible mechanism is a reduction of mitochondrial membrane potential (Quintanilla et al., 2008) which can lead to mitochondrial dysfunction, opening of mitochondrial permeability transition pores, cytochrome *c* release and apoptosis (Zorov et al., 2009). To this end PPARγ activation has been shown to upregulate Bcl-2 (Fuenzalida et al., 2007), an anti-apoptotic protein which inhibits the opening of mitochondrial permeability transition pores (Zorov et al., 2009). Alternatively inhibition of PPARγ may promote or exacerbate a harmful pro-inflammatory response as PPARγ upregulation is known to have anti-inflammatory response (Delerive et al., 2001; Desvergne and Wahli, 1999; Devchand et al., 1996). It will be interesting to see which mechanisms are involved but, given the complexity of PPARγ functions, it is likely to be a combination of many different pathways.

In conclusion, there is growing evidence that some of the cellular protective effects of PPARγ agonists do not require the activation of PPARγ (Chintharlapalli et al., 2005; Davies et al., 2001; Wang et al., 2011; Thal et al., 2011). The current study has extended these findings to the MPTP/MPP^+^ model of PD and suggests that prevention of oxidative stress is an important mechanism underlying the protective effect of rosiglitazone, but one that is independent of PPARγ activation. However, further studies using genetic manipulation strategies are needed to confirm these effects are independent of PPARγ activation. It also suggests that the glitazones as a drug class may impact upon the conversion of MPTP to its active metabolite MPP^+^ and that the protective effects attributed to this drug class may need careful reassessment. It is also worth noting that rosiglitazone has been removed from the European market as an antidiabetic due to cardiovascular toxicity and pioglitazone due to increasing the risk of bladder cancer (EMA/CHMP/586211/2010), affecting their consideration as useful translational drugs for the treatment of PD. This study has also demonstrated that PPARγ is important for neuronal survival as GW9662 was toxic to neurons. The results of this study add weight to there being a protective role for PPARγ agonists against MPTP toxicity, but also highlight the need for caution when manipulating PPARγ by pharmacological means.

## Figures and Tables

**Table 1:** Primer sequences and annealing temperatures used for quantitative-PCR.

Target	Forward primer (5′–3′)	Reverse primer (5′–3′)	Annealing temperature (°)
Human SOD1	AAGGCCGTGTGCGTGCTGAA	TGCCCAAGTCTCCAACATGCCT	64
Human GSTπ	GCTCACTCAAAGCCTCCTGCCT	AGTCATCCTTGCCCGCCTCA	64
Human β-actin	TGGTGGGCATGGGTCAGAAGGATT	AGGGATAGCACAGCCTGGATAGCA	64
Mouse PPARγ	GAGCTGACCCAATGGTTGCTG	GCTTCAATCGGATGGTTCTTC	62
Mouse β-actin	TGTGATGGTTGGGAATGGGTCAG	TTTGATGTCACGCACGATTTCC	67

PPARγ — peroxisome proliferator-activated receptor γ; SOD1 — superoxide dismutase 1; GST — glutathione-S-transferase.

**Table 2:** Effect of rosiglitazone and GW9662 on striatal levels of MPP^+^. Rosiglitazone reduced striatal MPP^+^ levels compared to mice receiving vehicle (5% DMSO v/v). No differences were seen in striatal levels of MPP^+^ between mice infused with GW9662 or those receiving vehicle. Data are mean ± SEM, n = 4 mice per group. *p < 0.05 compared to vehicle treated group (Kruskal–Wallis test with Mann Whitney U-post hoc tests).

	Vehicle	Rosiglitazone	GW0742
MPP^+^ (μg/g wet tissue weight)	12.31 ± 2.90	3.42 ± 0.70*	11.66 ± 3.46

**Table 3:** Effect of GW9662 treatment on striatal dopamine and DOPAC levels. No difference is seen in MPTP induced reductions in dopamine and DOPAC levels between mice treated with GW9662 (5 mg/kg daily) or those receiving vehicle (5% DMSO — see text for details). Data are mean ± SEM, n = 4–5 mice per group. *p < 0.05, **p < 0.01, ***p < 0.001 compared to appropriate saline treated group (Kruskal–Wallis test with Mann Whitney U-post hoc tests).

	Saline		MPTP	
Vehicle	GW9662	Vehicle	GW9662
Dopamine (ng/mg wet tissue)	11.81 ± 1.80	14.01 ± 1.40	1.13 ± 0.30*	1.00 ± 0.45***
DOPAC (ng/mg wet tissue)	1.62 ± 0.38	1.41 ± 0.22	0.25 ± 0.10**	0.22 ± 0.04***
